# Androgen Stimulates Growth of Mouse Preantral Follicles *In Vitro*: Interaction With Follicle-Stimulating Hormone and With Growth Factors of the TGF*β* Superfamily

**DOI:** 10.1210/en.2016-1538

**Published:** 2017-01-24

**Authors:** Mhairi Laird, Kacie Thomson, Mark Fenwick, Jocelyn Mora, Stephen Franks, Kate Hardy

**Affiliations:** Institute of Reproductive and Developmental Biology, Imperial College London, Hammersmith Hospital, London W12 0NN, United Kingdom

## Abstract

Androgens are essential for the normal function of mature antral follicles but also have a role in the early stages of follicle development. Polycystic ovary syndrome (PCOS), the most common cause of anovulatory infertility, is characterized by androgen excess and aberrant follicle development that includes accelerated early follicle growth. We have examined the effects of testosterone and dihydrotestosterone (DHT) on development of isolated mouse preantral follicles in culture with the specific aim of investigating interaction with follicle-stimulating hormone (FSH), the steroidogenic pathway, and growth factors of the TGF*β* superfamily that are known to have a role in early follicle development. Both testosterone and DHT stimulated follicle growth and augmented FSH-induced growth and increased the incidence of antrum formation among the granulosa cell layers of these preantral follicles after 72 hours in culture. Effects of both androgens were reversed by the androgen receptor antagonist flutamide. FSH receptor expression was increased in response to both testosterone and DHT, as was that of *Star*, whereas *Cyp11a1* was down-regulated. The key androgen-induced changes in the TGF*β* signaling pathway were down-regulation of *Amh*, *Bmp15*, and their receptors. Inhibition of *Alk6* (*Bmpr1b*), a putative partner for *Amhr2* and *Bmpr2*, by dorsomorphin resulted in augmentation of androgen-stimulated growth and modification of androgen-induced gene expression. Our findings point to varied effects of androgen on preantral follicle growth and function, including interaction with FSH-activated growth and steroidogenesis, and, importantly, implicate the intrafollicular TGF*β* system as a key mediator of androgen action. These findings provide insight into abnormal early follicle development in PCOS.

## Teaser

We examined androgen effects on mouse preantral follicles in culture. Testosterone and DHT stimulated growth, augmented FSH action, and induced significant changes in the TGFβ signaling pathway.

It has long been recognized that androgens have a physiological role in normal ovarian function. Androgens provide an obligatory substrate for estradiol production by maturing antral follicles [and may enhance either basal or follicle-stimulating hormone (FSH)–induced steroidogenesis by isolated granulosa cells (GCs)] (1–4), but there is also evidence that androgens may be necessary for the earliest stages of ovarian follicle development. Mice lacking a functional androgen receptor (AR) have impaired fertility (reduced litter size and/or reduced reproductive lifespan) but also show impaired growth and enhanced atresia of preantral follicles (5–7). Both aromatizable and nonaromatizable androgens have been shown to stimulate growth of isolated mouse preantral follicles (1) and effect activation of follicle development in fragments of bovine ovarian cortex (8, 9).

Exposure to excess androgen, however, is associated with ovarian dysfunction. In experimental animals, androgens inhibit proliferation (10) and increase apoptosis (11) in GCs from mature rat follicles. Importantly, ovarian dysfunction is a major feature of women with polycystic ovary syndrome (PCOS), a very common endocrine disorder in which ovarian hyperandrogenism is the key biochemical feature (12, 13). Infrequent or absent ovulation is characteristic, and this is associated with arrest of antral follicles during the final stages of maturation (14). However, anovulation in PCOS is also associated with aberrant development of preantral follicles, the key features of which are increased activation of follicle growth from the primordial stage (15) and enhanced GC proliferation in small preantral follicles (16) coupled with apparent “stockpiling” of follicles at the primary stage (17). Thus, ovarian dysfunction in PCOS would seem to have its origins at the earliest stages of follicle development at a point when, under physiological conditions, gonadotropin action is not obligatory and local growth factors are likely to play an important role. Furthermore, the abnormalities observed in preantral follicle development are characterized by enhanced rather than impaired activation and growth, and there is evidence that androgens may play a part.

The prenatally androgenized (PA) sheep is a well-established large animal model of PCOS. Lambs born to ewes that have been exposed during pregnancy to large doses of exogenous testosterone or dihydrotestosterone (DHT) have both reproductive and metabolic abnormalities that are reminiscent of PCOS (18, 19). Critically, ovarian dysfunction includes not only evidence of disrupted neuroendocrine control of the ovulation cycle but also abnormal preantral follicle development. In particular, the pattern of early follicle development in the ovaries of the PA sheep mirrors that observed in ovarian tissue from women with PCOS; the proportion of growing follicles is increased, and the primordial follicle population reciprocally diminished in ovary cortex from PA compared with control animals (20, 21).

We have previously used isolated mouse preantral follicles in culture to examine the direct effects, on early developing follicles, of growth factors on follicle growth, GC proliferation and gene expression (22, 23). Here we have applied this methodology to the investigation of the effects of androgens with the specific aims of investigating the interaction of androgens with FSH and with growth factors of the TGF*β* superfamily. These growth factors have a key role in ovarian follicular function, and our previous studies of isolated mouse preantral follicles have provided evidence for the involvement of both inhibitory and stimulatory TGF*β* molecules (and their endogenous inhibitors and binding proteins) in growth and function of small preantral follicles (22, 23).

## Material and Methods

### Tissue collection, follicle isolation, and culture

Whole ovaries were collected from C57BL/6 female mice aged 15 to 16 and 28 days postpartum (Harlan, Shardlow, United Kingdom). Mice were housed in accordance with the Animals (Scientific Procedures) Act of 1986 and associated Codes of Practice. Ovaries were removed, and those from 28-day-old mice were fixed in 10% neutral buffered formalin (Sigma Aldrich Company, Dorset, United Kingdom). Preantral follicles were mechanically isolated from mice aged 15 to 16 days using acupuncture needles, as previously described (22, 23), and placed in Liebovitz L15 medium (Life Technologies, Paisley, United Kingdom) supplemented with 1% (weight/volume) bovine serum albumin (Sigma). Individual follicles were then transferred into a single well (one follicle per well) in a 96-well plate containing 100 µL Minimal Essential Medium alpha (MEM-*α*, Life Technologies) supplemented with 0.1% bovine serum albumin, 75 µg/mL penicillin (Sigma), 100 µg/mL streptomycin sulfate (Sigma), and a cocktail of 5 µg/mL insulin, 5 µg/mL transferrin, and 5 ng/mL sodium selenite (Sigma). Isolated follicles from each ovary were distributed randomly (by picking up from a slightly out-of-focus drop of culture medium) and evenly between treatments in a single 96-well plate. Up to six ovaries (plates) were cultured per experiment. Follicles were incubated in a humidified incubator in 5% CO_2_ at 37°C for up to 72 hours.

### Analysis of follicle development

Follicles were photographed daily, and follicle area or diameter was measured using ImageJ 1.45s (https://imagej.nih.gov/ij/) at each time point. At the end of culture period, follicles were either snap frozen for expression studies or fixed in formalin for immunofluorescence studies.

**Figure 1.**
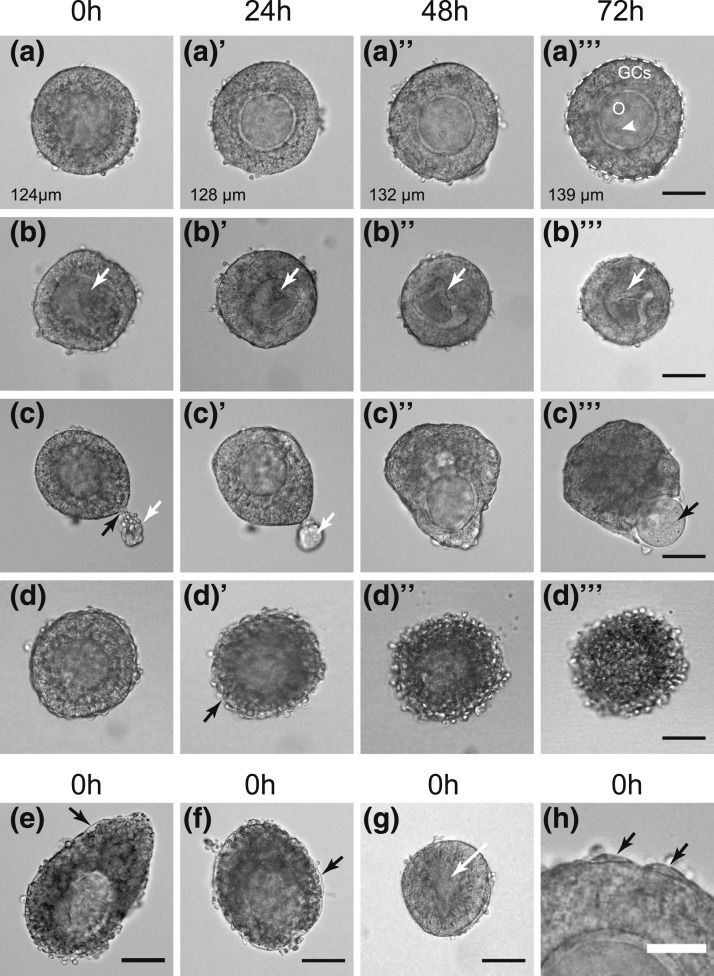
Morphology of manually isolated follicles that were included in the analysis (a; 84%) or excluded from analysis because of damage or atresia at the start of culture (b, c, e, f, and g; <15%) or onset of atresia during culture (d; <1%). (a) Healthy follicle cultured under control conditions (72 hours) with a central oocyte (O) and even layer of GCs. Follicle diameter shown in each image. The white dotted line (a’’’) indicates the basal lamina surrounding the GCs and shows the area that is measured. A nucleolus is arrowed (white arrowhead). Note the lack of theca cells on the basal lamina. (b) Follicle with indistinct oocyte (white arrow) that becomes increasingly misshapen and shrunken with time. (c) Follicle with damaged basal lamina (black arrow), resulting in extrusion of GCs (white arrow). With time in culture, the oocyte is extruded through the breach (black arrow). (d) Follicle that appears healthy at 0 hours but becomes atretic from 24 hours, with rounding up and progressive darkening of the GCs and loss of the basal lamina. (e and f) Atretic follicles at the start of culture (0 hours) with a detached basal lamina (black arrows) and darkened GCs. (g) Follicle with collapsing, indistinct oocyte (white arrow, 0 hours). (h) Sparse theca cells (black arrows) on basal lamina. Scale bars = 50 µm (a–g) and 25 µm (h).

During the course of this study, images and growth data on 1307 follicles were acquired. These images, taken at 24-hour intervals, as well as measurements were imported into a custom-made database (FileMaker Pro 11.v2; http://www.filemaker.com). This allowed automatic calculation of cumulative follicle growth in terms of relative size compared with the start of culture (time 0) for each follicle, as well as follicle diameter. The database also facilitated inspection of follicles during development, annotation of morphological features, and the implementation of strict inclusion and exclusion criteria. Follicle images were scored by one observer in a database layout that lacked information about treatment. Follicles with a central spherical oocyte and intact layer of GCs were included in the analysis [Fig. 1(a)]. Follicles were excluded from analysis if, at the start of culture, their oocyte was misshapen [Fig. 1(b) and 1(g)] or extruded from the follicle, if the follicle was atretic (darkened GCs) [Fig. 1(e) and 1(f)], or if the basal lamina was damaged with the oocyte being subsequently extruded from the follicle during culture [Fig. 1(c)]. Follicles were also excluded from analysis if they died during culture, with oocyte extrusion or the onset of atresia [Fig. 1(d)].

The database also expedited search and export of data for follicles cultured under the same conditions for several further investigations, providing an extended dataset for analysis of developmental and morphological features such as antrum formation or for comparing development of follicles of differing sizes in various treatments.

### Effect of androgens on preantral follicle development

Isolated follicles were cultured in either control medium or with androgen (either 10 nM DHT or 10 nM testosterone; Sigma) in the presence and absence of 20 µM flutamide (a competitive AR inhibitor; Sigma). DHT, testosterone, and flutamide were dissolved in ethanol, and the final concentration of ethanol in each treatment and in control wells was 0.1%.

### Effect of initial follicle size on responsiveness to DHT

Upon completion of all the culture experiments, the database was mined for follicles cultured under control conditions and in the presence of DHT alone. Follicles were grouped in five bins according to initial diameter at 0 hours: <100, 100 to 109.99, 110 to 119.99, 120 to 129.99, and ≥130 µm in diameter. The overall growth of follicles in the presence and absence of DHT was compared using linear regression (Prism 6 for Mac OS X, version 6.0a; http://www.graphpad.com).

### Effect of DHT on AR expression

Mechanically isolated follicles cultured for 24 or 72 hours in the presence or absence of 10 nM DHT were formalin fixed and set in 2% (weight/volume) low-melting-point agarose (Sigma) before being dehydrated through an alcohol series, embedded in wax, and serially sectioned (5 µm) onto Superfrost charged slides (VWR, Lutterworth, United Kingdom). Immunofluoresence detection of AR was carried out as described later, and one central section of each follicle was selected for analysis. Confocal images of individual follicles were imported into ImageJ and split into red-green-blue channels. Blue 4′,6-diamidino-2-phenylindole (DAPI)–labeled nuclei were thresholded (same threshold value for all follicles) to produce a binary image (white DAPI, positive; black DAPI, negative) and define the nuclei. Nuclei were outlined using the “Analyze Particles” command. In the green channel, AR labeling was thresholded (with the same threshold maintained for all follicles), the nuclear outlines were overlaid, and the area of labeling exceeding the threshold was measured within the nuclear outlines, and for the GC layer as a whole. The proportion of nuclear area or GC area that exceeded the threshold (*i.e.*, the proportion of the nuclei, or GC layer, that was AR positive) was compared for control and DHT-treated follicles at 24 and 72 hours.

### Antrum formation

The database was further used for investigating antrum formation in the presence and absence of DHT and under other treatment conditions. An antrum was clearly visible as a translucent area with a clear margin in the GCs. Images were inspected and annotated for appearance of an early antrum by 72 hours of culture.

### Effect of androgen and FSH on preantral follicle growth

Follicles were treated with control medium (including 0.1% ethanol vehicle) or with 10 nM DHT and/or 10 ng/mL FSH (recombinant human FSH; National Hormone and Peptide Program, National Institute of Diabetes and Digestive and Kidney Diseases, Dr. A.F. Parlow, Torrance, CA). Follicles were maintained in culture for 24, 48, or 72 hours before being frozen for gene expression studies as previously described. Images of each individually cultured follicle were taken at 0, 24, 48, and 72 hours, as appropriate.

### Effect of inhibiting Alk6 on DHT action

Dorsomorphin (DSM; Sigma) was used as an inhibitor of the type I TGF*β* receptors ALK2, ALK3, and ALK6 (24). Follicles were cultured with vehicle alone, 10 nM DHT alone, DHT and 1 µM DSM (23), and DSM alone for 72 hours. Follicle growth was assessed as described previously. At the end of culture, follicles were snap frozen in liquid nitrogen for gene expression studies, as described later.

### Expression studies

At the end of each appropriate culture period, approximately five follicles per treatment group were pooled into one tube and snap frozen in liquid nitrogen. Samples were then processed for RNA extraction using RNeasy microcolumns as per manufacturer’s instructions (Qiagen, Manchester, United Kingdom). The resulting RNA was concentrated using vacuum centrifugation to provide a final volume of approximately 5 µL and converted to complementary DNA (cDNA) using SuperScript III reverse transcription and random hexamers, according to manufacturer’s instructions (Life Technologies). cDNA products were used for quantitative polymerase chain reaction as previously described (23). Briefly, primers were designed using Primer3 plus v2.3.6 software (Table 1). cDNA or water (control) was added to a reaction mix that included 500 nM of appropriate primers (ROX, KAPA SYBR FAST; KAPA Biosystems Limited, London, United Kingdom) and nuclease-free water. Each sample or control mix was plated in duplicate, and amplification was carried out using an Applied Biosystems 7900HT Fast instrument. A melt curve analysis was carried out on each sample. *Atp5b* (Primer Design, Southampton, United Kingdom) was determined to be an appropriate internal reference gene and therefore used in this series of studies. Expression levels were normalized to the internal reference gene and calculated as fold changes relative to the untreated control group, using the 2-delta-delta-cycle threshold method (25).

**Table 1.** **Primers Used for Quantitative Polymerase Chain Reaction Assays**

Gene Symbol	Primer Sequence (5′-3′)	GenBank Accession	Product Size (bp)
*Amh*	Fwd: ggggcacacagaacctct	NM_007445.2	124
	Rev: gcaccttctctgcttggttg		
*Bmp15*	Fwd: gagaaccgcacgattggag	NM_009757.4	134
	Rev: agttcgtatgctacctggtttg		
*Gdf9*	Fwd: tcacctctacaataccgtccgg	NM_008110.2	139
	Rev: gagcaagtgttccatggcagtc		
*Fshr*	Fwd: acaactgtgcattcaacggaac	NM_013523.3	187
	Rev: gacctggccctcaacttctt		
*Ar*	Fwd: attctggatgggactgatg	NM_013476.4	246
	Rev: gcccatccactggaataatg		
*Star*	Fwd: aagaacaacccttgagcacct	NM_011485.4	267
	Rev: ctccctgctggatgtaggac		
*Cyp11a1*	Fwd: ctgggcactttggagtcagt	NM_019779.3	185
	Rev: aggacgattcggtctttcttc		
*Amhr2*	Fwd: acagcatgaccatatcgttcg	NM_144547.2	122
	Rev: gagtcaagtagtggcataaggag		
*Bmpr2*	Fwd: actgggaggtgtttatgaggtg	NM_007561.4	150
	Rev: ggaacttgggtctctgcttct		
*Alk3* (*Bmpr1a*)	Fwd: tgactttagcaccagaggatacc	NM_009758.4	177
	Rev: cagagccttcatacttcatacacc		
*Alk4* (*Acvr1b*)	Fwd: caacatgaagcactttgactcc	NM_007395.3	177
	Rev: tcacatacaacctttcgcatct		
*Alk5* (*Tgfbr1*)	Fwd: attgctccaaaccacagagtag	NM_009370.3	157
	Rev: caccaatagaacagcgtcgag		
*Alk6* (*Bmpr1b*)	Fwd: aggaggatggagagagtacagc	NM_007560.4	166
	Rev: ccagaggtgacaacaggcatt		

**Table 2.** **Antibody Table**

Peptide/Protein Target	Antigen Sequence	Name of Antibody	Manufacturer, Catalog No., RRID No.	Species Raised in Monoclonal or Polyclonal	Dilution Used	RRID
Androgen receptor	Epitope mapping at N terminus	AR (*N*-20)	Santa Cruz Biotechnology, sc-816	Rabbit, polyclonal	1 in 200	AB_1563391
Ki67	Recombinant protein specific to the amino terminus of Ki-67 protein	Ki-67 (D3B5; mouse preferred; immunohistochemistry formulated)	Cell Signaling Technology, 12202	Rabbit, monoclonal	1 in 200	AB_2620142
Cleaved caspase-3	Large fragment (17/19 kDa) resulting from cleavage adjacent to Asp175	Cleaved caspase-3 (Asp175; 5A1E)	Cell Signaling Technology, 9664	Rabbit, monoclonal	1 in 200	AB_2070042

Abbreviation: RRID, Research Resource Identifier.

### Immunofluorescence studies

Whole formalin-fixed, paraffin-embedded ovaries were serially sectioned (5 µm) to examine AR protein expression. For examination of the effect of treatment on AR protein expression, cell proliferation, and apoptosis, formalin-fixed, cultured follicles were set in 2% (weight/volume) low-melting-point agarose (Sigma) before being dehydrated through an alcohol series, embedded in wax, and serially sectioned (5 µm) onto Superfrost charged slides (VWR, Lutterworth, United Kingdom). Ovary and follicle sections were dewaxed and rehydrated prior to boiling in citrate buffer (10 mM citric acid, pH 6.0) and blocking of nonspecific binding within 20% normal goat serum (Sigma). After this, with no washing step, sections were incubated in rabbit anti-mouse AR antibody NR-20 (sc-816, 1:200 dilution; Santa Cruz, Dallas, TX), rabbit anti-mouse D3B5 Ki67 (1:200; New England Biolabs, Hitchin, United Kingdom), or rabbit anti-mouse cleaved caspase-3 (1:200; 9664, Cell Signaling) diluted in 2% normal goat serum (Table 2). The latter were subsequently exposed to a 1:9 ratio of terminal deoxynucleotidyltransferase-mediated dUTP nick end labeling (TUNEL) enzyme:label (Roche) for 60 minutes at 37°C after phosphate-buffered saline washes. The negative control for AR immunofluorescence was rabbit immunoglobulin G (Sigma i8140) at the same concentration as that used for the primary antibody (0.5 µg/mL). The primary antibodies were incubated overnight at 4°C and then detected using an appropriate goat anti-rabbit Alexafluor secondary antibody (1:500; Life Technologies). All unstained nuclei were then visualized by incubation with DAPI (1:1000; Life Technologies) before sections were mounted under coverslips using Prolong Gold mounting medium containing DAPI (Life Technologies). Sections were visualized (×63 objective) and captured using a Leica inverted SP5 confocal laser-scanning microscope (Leica Microsystems, Cambridge, United Kingdom).

Ki67-positive, caspase-positive, TUNEL-positive, and unlabeled DAPI-stained cells or nuclei were counted using the ImageJ plugin “cell counter.jar” (https://imagej.nih.gov/ij/plugins/cell-counter.html) within ImageJ (1.45s). DAPI-labeled (blue), Ki67-positive (green), and TUNEL-positive (green) nuclei or caspase-positive (red) cells were counted in a single midsection of the follicle, and the proportion of Ki67-positive, caspase-positive, or TUNEL-positive nuclei/cells was calculated.

### Statistics

The relative area of follicles in different treatments (area at time_x_/area at time_0_, where time_x_ = 24, 48, or 72) was compared at each time point using one-way analysis of variance (ANOVA) with the appropriate *post hoc* test for multiple comparisons (Prism 6). Gene expression values were log_2_ transformed, and the difference between treatments was analyzed using a *t* test. In all cases, a *P* value less than 0.05 was considered statistically significant.

## Results

### Follicle culture

In this study, a total of 1307 follicles from 45 ovaries were cultured in various conditions. On average, 29 ± 0.68 follicles (mean and standard error of the mean [SEM]; range 22 to 40) per ovary were cultured and evenly distributed between treatments. Overall, less than 15% of follicles placed in culture were found, upon closer high-power inspection, to be either atretic or damaged at the outset (Fig. 1) and were therefore excluded from further analysis. A very small number of follicles (12) died during culture: four controls, three cultured in DHT alone and five in DHT plus FSH.

### Effect of androgens on preantral follicle growth

**Figure 2.**
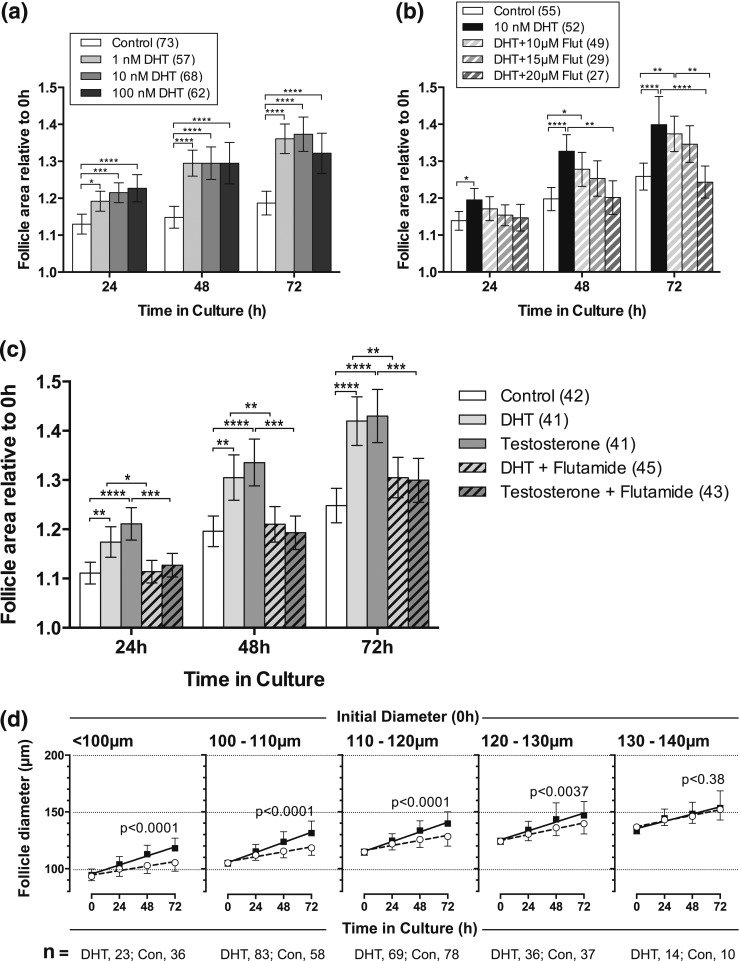
Follicle growth *in vitro* in response to DHT and testosterone. (a) Response of individual follicles to increasing concentrations of DHT. The relative area of follicles in different treatments (area at time_x_/area at time_0_, where time_x_ = 24, 48, or 72) was compared at each time point using one-way ANOVA with a Tukey’s multiple comparisons test. *P < 0.05, ***P < 0.001, ****P < 0.0001. Values are mean and 95% confidence interval. Numbers in parentheses are numbers of follicles. (b) Response of follicles to DHT (10 nM) in the presence of increasing doses of the AR inhibitor flutamide (Flut). Values are mean and 95% confidence interval. Statistical analysis as in a. *P < 0.05, **P < 0.01, ****P < 00001. (c) Response of follicles to DHT (10 nM) or testosterone (10 nM) in the absence and presence of flutamide (20 μM). DHT- and T-stimulated follicle growth was reversed by flutamide. Values are mean and 95% confidence interval. Statistical analysis as in a. *P < 0.05, **P < 0.01, ***P < 0.001, ****P < 00001. (d) Effect of DHT on *in vitro* growth of follicles of different initial sizes. Values are means and standard deviation, and lines are regression slopes. Slopes were compared using linear regression. Follicles larger than 130 µm in diameter at the start of culture did not grow more in the presence of DHT. Con, control.

DHT at doses of between 1 and 100 nM resulted in an increase in follicle growth compared with vehicle alone at 24, 48, and 72 hours in culture [Fig. 2(a)]. Although the largest response was achieved with a dose of 100 nM, there was little difference between the effects of the different doses. A similar dose-response range was found for the effects of testosterone on follicle growth (data not shown). In all subsequent studies, a dose of 10 nM DHT or testosterone (approximating to physiological serum concentrations of testosterone) was therefore used.

Flutamide inhibited DHT-induced follicle growth in a dose-dependent manner [Fig. 2(b)], but as the effect was significant only at a flutamide concentration of 20 μM, this dose was used in further studies

The effects of DHT (a nonaromatizable androgen) and testosterone (an aromatizable androgen) were compared. Both androgens significantly increased follicle growth over 72 hours in culture [Fig. 2(c)]. At all time points, DHT and testosterone did not significantly differ in their stimulatory effect on follicle growth. The addition of flutamide to androgen-treated follicles inhibited the effect of both androgens and brought follicle growth back in line with controls.

We were interested in whether the responsiveness of preantral follicles to DHT changed with follicle size. Taking an overview of all the data that we collated in the database during these studies, we explored how DHT affected the growth of follicles of different sizes. Isolated follicles placed in culture ranged in size from 75 to 150 µm in diameter. Intriguingly, although smaller follicles grew significantly more in the presence of DHT, there was no significant increase in the growth of larger follicles (>130 µm) with DHT. This suggests that these follicles have become less androgen responsive [Fig. 2(d)].

### Expression of AR in whole mouse ovary and in cultured follicles

**Figure 3.**
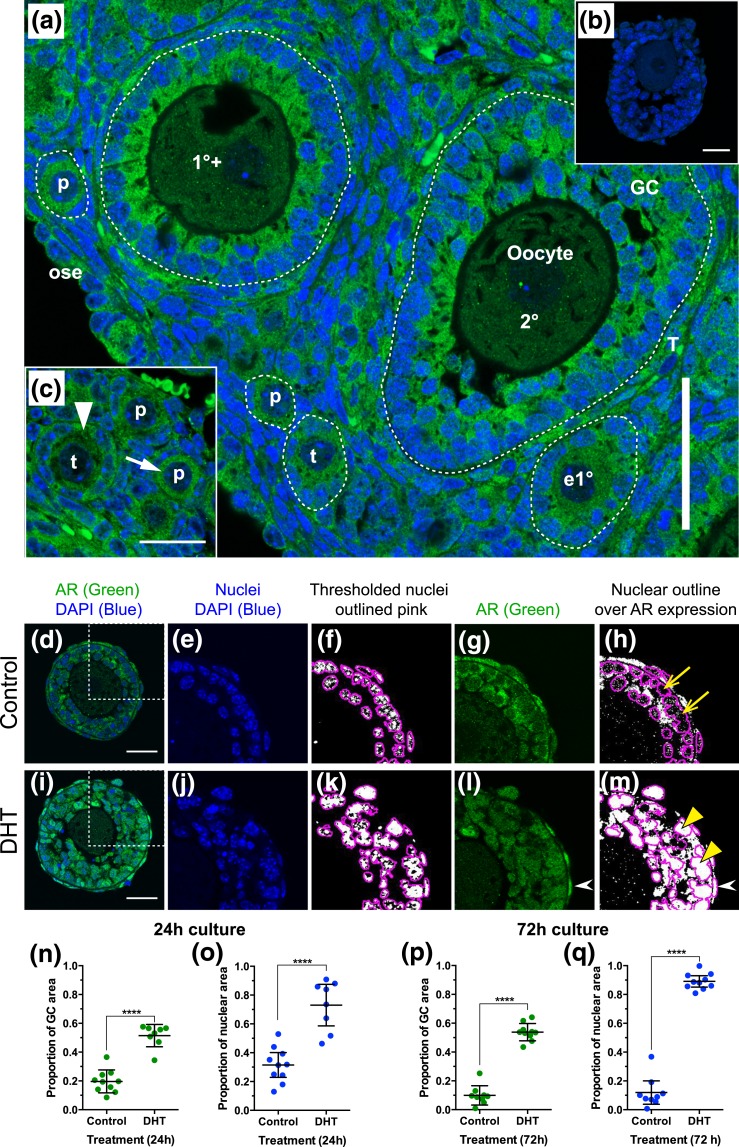
AR is present in oocytes, GCs, and theca cells of preantral follicles, and overall expression and nuclear localization of AR increases in the presence of DHT. (a) AR expression (green) in mouse prepubertal mouse ovary on day 17 postpartum. DAPI-labeled nuclei are blue. Scale bar = 50 µm. White dotted lines indicate basal lamina surrounding follicles. (b) Rabbit immunoglobulin G control. Scale bar = 25 µm. (c) Strong labeling of ooplasm (white arrow) and GC cytoplasm (white arrowhead) in primordial and transitional follicles. Scale bar is 25 µm. (d–m) Quantification of AR in formalin-fixed, paraffin-embedded sections of follicles cultured in the absence (d–h) and presence (i–m) of DHT. (d and i) Confocal image of follicle showing expression of AR (green) and DAPI-labeled nuclei (blue). Scale bars = 25 µm. White dotted box defines enlarged images (e–h and j–m). Images were imported into ImageJ and split into red-green-blue channels. Blue DAPI-labeled nuclei (e and j) were thresholded (f and k; where white is DAPI positive and black DAPI negative), and nuclei were outlined using the “Analyze Particles” command (f and k). In the green channel, AR labeling (g and l) was thresholded (with the same threshold maintained for all follicles), the nuclear outlines were overlaid, and the area of labeling exceeding the threshold was measured within the nuclear outlines (h and m), and for the GC layer as a whole. Yellow arrows (h) indicate nuclei with little nuclear AR, and yellow arrowheads (m) indicate nuclei with strong nuclear AR. White arrowheads (l and m) show strong AR expression in theca cell nuclei. Values were plotted and compared between treatments using a Mann-Whitney test. Overall AR protein expression in the GC layer was significantly increased after 24 hours (n) and 72 hours (p) culture in the presence of DHT. Nuclear localization of AR was also significantly increased in the presence of DHT at 24 hours (o) and 72 hours (q). 1°+, primary stage with second layer of GCs appearing; 2°, secondary stage; e1°, early primary; ose, ovarian surface epithelium; p, primordial; T, theca; t, transitional.

Immunofluorescence for AR showed expression in follicles from the primordial stage onwards in day 17 ovary [Fig. 3(a)]. In primordial and transitional follicles, AR appeared to be expressed in the ooplasm more strongly than at later stages, and even in very early follicles, AR was clearly present in GCs [Fig. 3(c)].

Immunofluorescence labeling of AR in follicles cultured in the presence and absence of DHT for 24 and 72 hours was quantified in the GC layer, as well as specifically in the nuclei [Fig. 3(d–m)]. DHT treatment resulted in significantly increased expression of AR in the GCs following 24 hours [Fig. 3(n)] and 72 hours [Fig. 3(p)] of culture. Furthermore, translocation of AR to the nucleus was minimal in control follicles [Fig. 3(h)] but was significantly increased in follicles exposed to DHT at both time points [Fig. 3(m), 3(o), and 3(q)]. Strong labeling of theca cell nuclei for AR was also observed following DHT treatment [Fig. 3(l) and 3(m), white arrows].

### DHT and antrum formation

**Figure 4.**
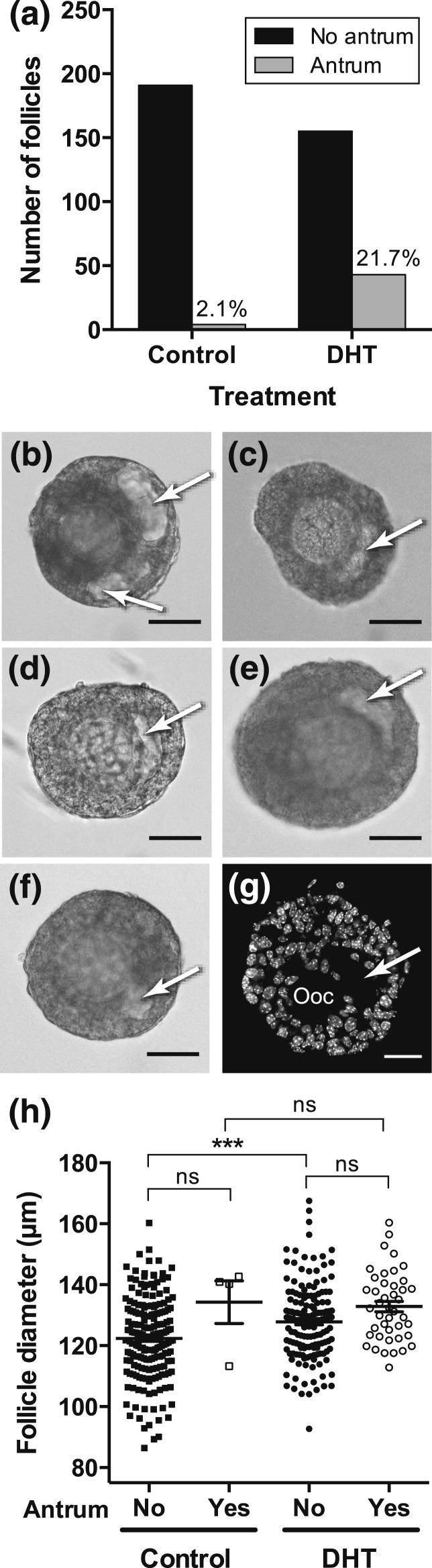
Follicles cultured in the presence of DHT had a 10-fold increased incidence of early antrum formation. (a) The number of follicles in the absence and presence of DHT that formed an antrum by 72 hours of culture. Numbers above the bars are the percentage of follicles that formed an antrum (*P* < 0.0001; two-sided Fisher’s exact test. (b­–f) Examples of preantral follicles with an antrum (arrowed, translucent area within the GCs) cultured in DHT. (g) Confocal image of DHT-treated follicle exposed to DHT *in vitro* with oocyte (ooc) and antrum arrowed. (h) Diameter of follicles at 48 hours (*i.e.*, at the onset of possible antrum formation) that did, or did not, have an antrum at 72 hours. Four statistical comparisons were made (to compare diameter of follicles with and without antra within a treatment; and of follicles with, or without, antra between treatments) using one-way ANOVA with a Sidak’s multiple comparison test. ***P < 0.001. ns, not significant.

Examination of 219 follicles cultured under control conditions and 216 follicles cultured with DHT alone that were identified from the database showed that significantly (*P* < 0.0001) more follicles cultured in DHT developed an antrum during 72 hours of culture [Fig. 4(a–g)]. We expected that this would be due to the increased follicle growth observed in the presence of DHT and that antra would be seen in the largest follicles. Surprisingly, we found that there was no significant difference in diameter between follicles with and without an antrum in the presence of DHT [Fig. 4(h)], suggesting that this developmental change is not due to accelerated growth but to another, as yet unknown, mechanism.

**Figure 5.**
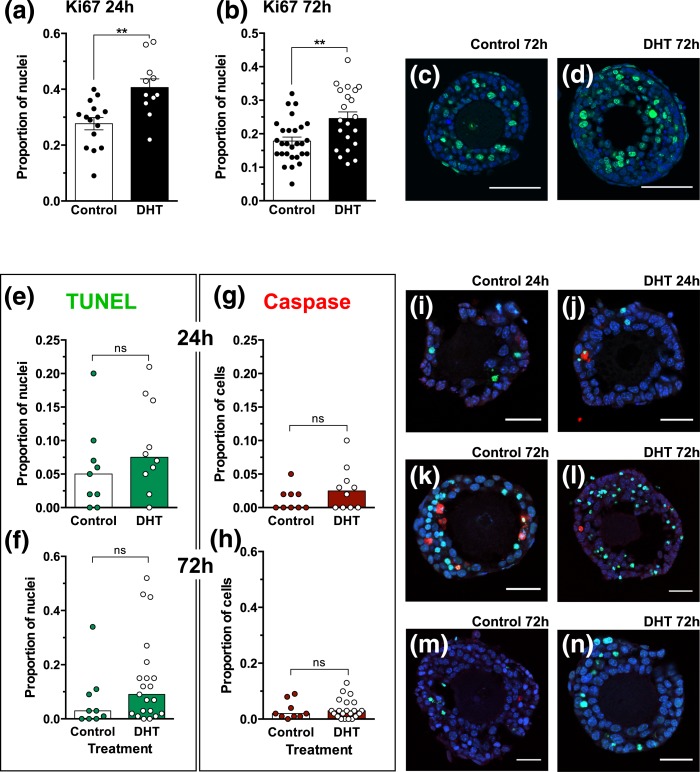
Follicles cultured in the presence of DHT had a greater proportion of Ki67-positive (green) nuclei than in control conditions after 24 hours (a) and 72 hours (b) of culture. Bars are means and 95% confidence interval; treatments were compared using an unpaired *t* test. **P < 0.01. (c and d) Confocal images of Ki-67–positive nuclei (green); scale bars = 50 µm. (e–h) There was no significant effect of treatment on TUNEL (e and f, green) and caspase (g and h, red) expression in control and DHT-treated follicles at 24 and 72 hours. Bars are medians; treatments were compared using a Mann-Whitney test. (i–g) Confocal images of TUNEL-labeled (green) and active caspase-positive GCs (red) in follicles at 24 hours (i and j) and 72 hours (k–n) cultured in the absence (i, k, and m) and presence (j, l, and n) of DHT. Nuclei were counterstained with DAPI (blue). Scale bars = 25 µm (i–n).

The stimulatory action of DHT on follicle growth was accompanied by increased protein expression of the proliferation marker Ki67 in GCs of preantral follicles following both 24 and 72 hours of culture [Fig. 5(a–d)]. Follicles were assessed after 24 and 72 hours of culture for evidence of apoptosis using dual labeling for TUNEL [Fig. 5(e) and 5(f)] and active caspase-3 [Fig. 5(g) and 5(h)]. There was no significant difference between control and DHT-treated follicles in the incidence of these apoptotic markers. Generally, apoptosis was observed in a small proportion of GCs at 24 hours [Fig. 5(i) and 5(j)] and 72 hours [Fig. 5(m) and 5(n)], with the exception of a few follicles showing more widespread TUNEL labeling [Fig. 5(k) and 5(l)].

### Interaction of DHT with FSH

**Figure 6.**
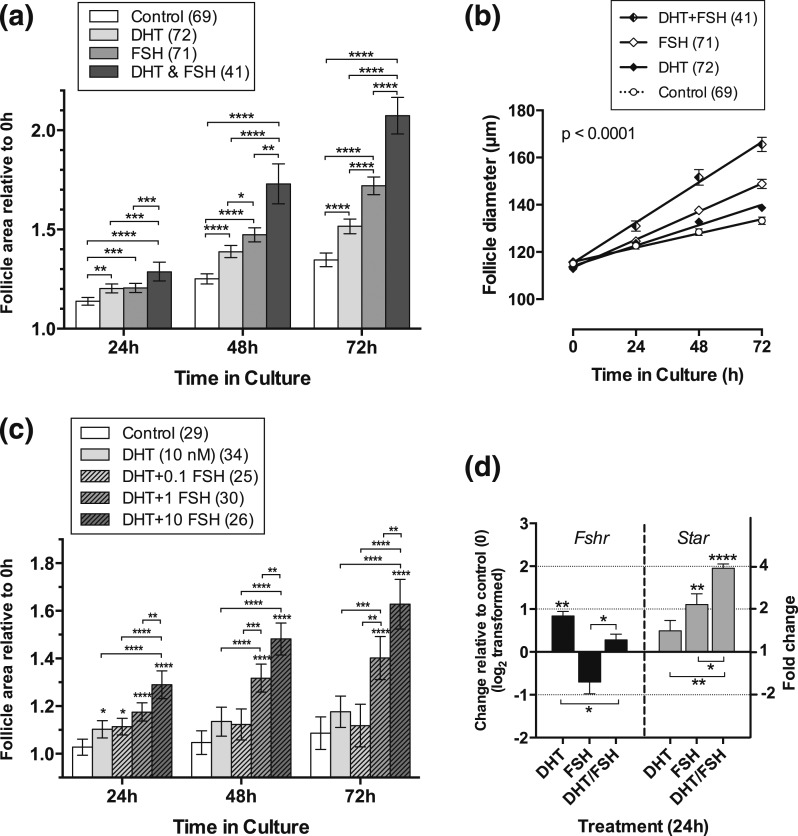
(a) FSH further stimulates DHT-stimulated follicle growth after 48 hours of culture. Relative area of follicles in different treatments was compared at each time point using one-way ANOVA with a Tukey’s multiple comparisons test. *P < 0.05, **P < 0.01, ***P < 0.001, ****P < 00001. Values are mean and 95% confidence interval. Numbers in parentheses are numbers of follicles. (b) Growth trajectory over time of follicles cultured in the absence and presence of DHT and/or FSH. A combination of DHT and FSH stimulates a greater rate of growth than DHT alone (*P* < 0.0001), FSH alone (*P* < 0.0001), or control (*P* < 0.0001). FSH or DHT alone stimulates a greater rate of growth than control (FSH, *P* < 0.0001; DHT, *P* < 0.002). The rate of growth in FSH alone is significantly greater than that in DHT alone (*P* < 0.0004). Values are means and SEM, and lines are regression slopes. Slopes were compared using linear regression. Numbers in parentheses are numbers of follicles. (c) Follicles cultured in the presence of DHT and increasing doses of FSH showed significant growth enhancement in the presence of 1 ng/mL and 10 ng/mL FSH, but not in the lowest dose of FSH (0.1 ng/mL). Statistical comparisons as in a. (d) Change in expression of *Fshr* and *Star* in four samples of five follicles, each cultured for 24 hours in the presence of DHT alone, FSH alone, or DHT and FSH in combination. Data have been log_2_ transformed, and treatments were compared with control (0) and between treatments using an unpaired *t* test. Fold change indicated in right-hand y axis. Asterisks by error bars indicate a significant difference in expression from control; differences between treatments are indicated by asterisks on horizontal lines. *P* values as in a. Values are mean and SEM.

FSH (10 ng/mL) significantly increased the proportionate growth of cultured preantral follicles compared with control. When DHT was added to the cultures, the combination of DHT and FSH increased follicle growth in comparison with either individual treatment alone after 24 hours and, particularly, after 48 and 72 hours in culture [Fig. 6(a)]. Interestingly, we observed that, after 72 hours in culture with the combined treatment of DHT and FSH (but not with the individual treatments alone), the basal lamina surrounding the GC layers in some follicles was disrupted, allowing extrusion of some GCs vertically as well as horizontally, and thereby possibly decreasing the precision of follicle measurement.

Overall, the rate of growth of follicles in the presence of both DHT and FSH was significantly greater than that in either treatment alone. Furthermore, follicles in treatments alone or in combination grew at a significantly faster rate than control follicles [Fig. 6(b)]. The dose of FSH used here was the lowest dose that elicited a maximal response in a previous study (26). We were interested to explore whether the presence of DHT would allow a lower dose of FSH to stimulate the same maximal response. This was not the case; lower doses of FSH resulted in minimal growth [Fig. 6(c)].

### Effects of androgens and FSH on gene expression

#### FSH receptor, steroid synthesis, and action

FSH receptor (*Fshr*) expression was significantly increased in follicles cultured in DHT compared with vehicle-treated controls after 24 hours in culture, whereas FSH treatment reduced expression of *Fshr*. This effect was partially reversed during cotreatment with DHT [Fig. 6(d)]. DHT alone had a small and insignificant effect on expression of *Star* but further enhanced FSH-stimulated *Star* expression.

**Figure 7.**
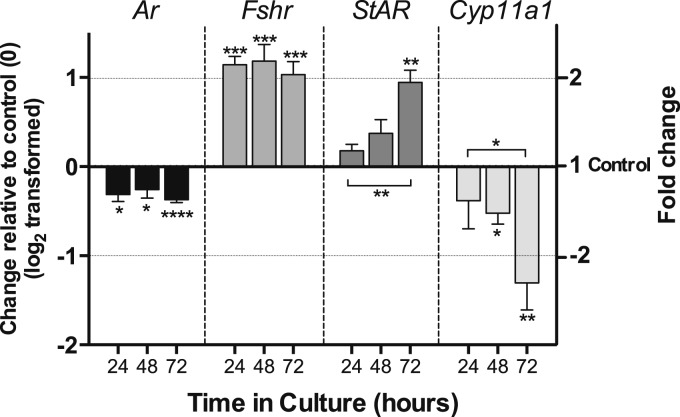
Time course of hormone receptor and steroidogenic enzyme gene expression in isolated follicles during 72 hours of culture in the presence of DHT. Change in expression of *Ar*, *Fshr1*, *Star*, and *Cyp11a1* in four to six samples of five follicles, each cultured for 24, 48, and 72 hours in the presence of 10 nM DHT. Data have been log_2_ transformed, and DHT treatment was compared with time-matched control samples (0) and between time points using an unpaired *t* test. Fold change is indicated in right-hand y axis. Asterisks by error bars indicate significant difference in expression from control, and differences between time points are indicated by asterisks on horizontal lines. *P < 0.05, **P < 0.01, ***P < 0.001, ****P < 00001. Values are mean and SEM.

We examined the time course of gene expression of androgen receptor (*Ar*), *Fshr*, *Star*, *Cyp11a1*, *Cyp17*, and *Cyp19* during 72 hours of culture in presence of DHT (Fig. 7). AR expression was consistently suppressed by DHT, whereas that of *Fshr* was increased at each time point. Expression of *Star* was significantly up-regulated after 72 hours in culture, whereas that of *Cyp11a1* was significantly reduced after 48 hours in culture and further reduced at 72 hours (Fig. 7). *Cyp19* expression was undetectable, as was that of *Cyp17*.

#### Effect of DHT and testosterone on TGF*β* ligands and receptors

**Figure 8.**
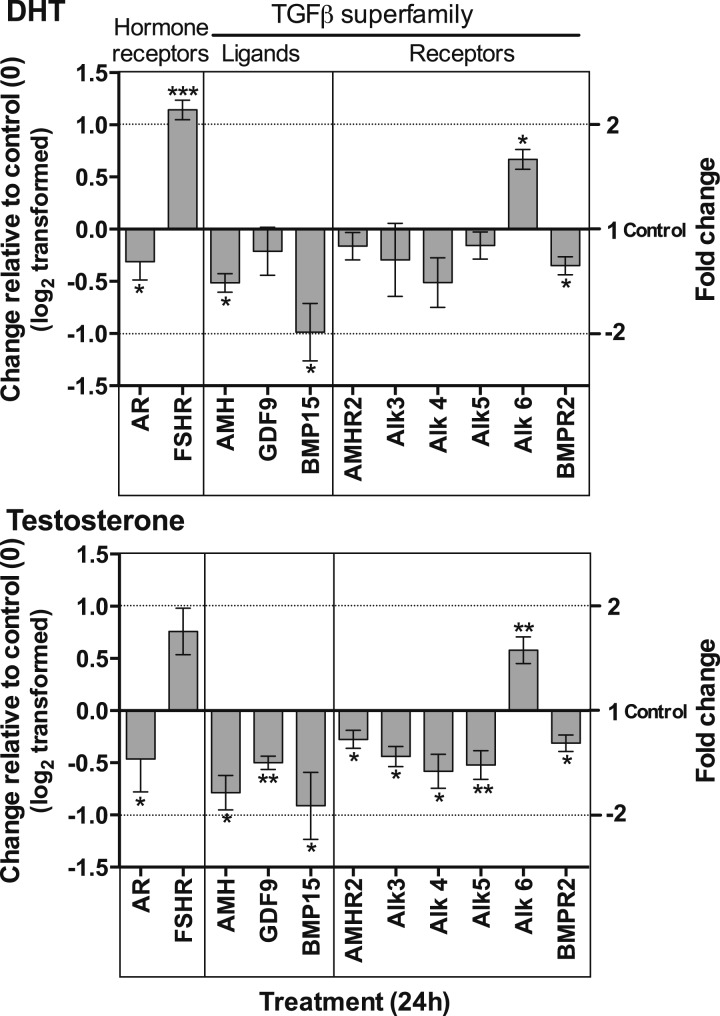
Effect of DHT (a) and testosterone (b) on gene expression of hormone receptors and members of the TGF*β* superfamily (ligands and receptors). Isolated follicles were cultured in the presence and absence of DHT or testosterone for 24 hours. Data have been log_2_ transformed, and DHT or testosterone treatment was compared with control samples (0) using an unpaired *t* test. Fold change is indicated on right-hand y axis. Asterisks by error bars indicate significant difference in expression from control. *P < 0.05, **P < 0.01, ***P < 0.001. Values are mean and SEM.

In this series of studies, we examined the effects of either DHT or testosterone on gene expression after 24 hours in culture (Fig. 8). Expression of *Ar* was reduced and that of *Fshr* increased by DHT, as shown previously; similar changes were seen in response to testosterone.

We observed a significant interaction of androgens with ligands and receptors of the TGF*β* superfamily in these studies (Fig. 8). Exposure to androgens during culture resulted in reduction of expression of *Amh* and *Bmp15* by both DHT and testosterone. A similar trend was observed for *Gdf9*, but the change was not significant in response to DHT. Testosterone, but not DHT, reduced expression of *Amhr2*, and both androgens reduced *Bmpr2* expression. Expression of the type 1 receptors *Alk3*, *Alk4*, and *Alk5* were similarly reduced by both androgens, but, again, only the actions of testosterone were significant. However, both DHT and testosterone stimulated expression of *Alk6*. All androgen-induced changes in gene expression were negated by coincubation with flutamide (data not shown).

**Figure 9.**
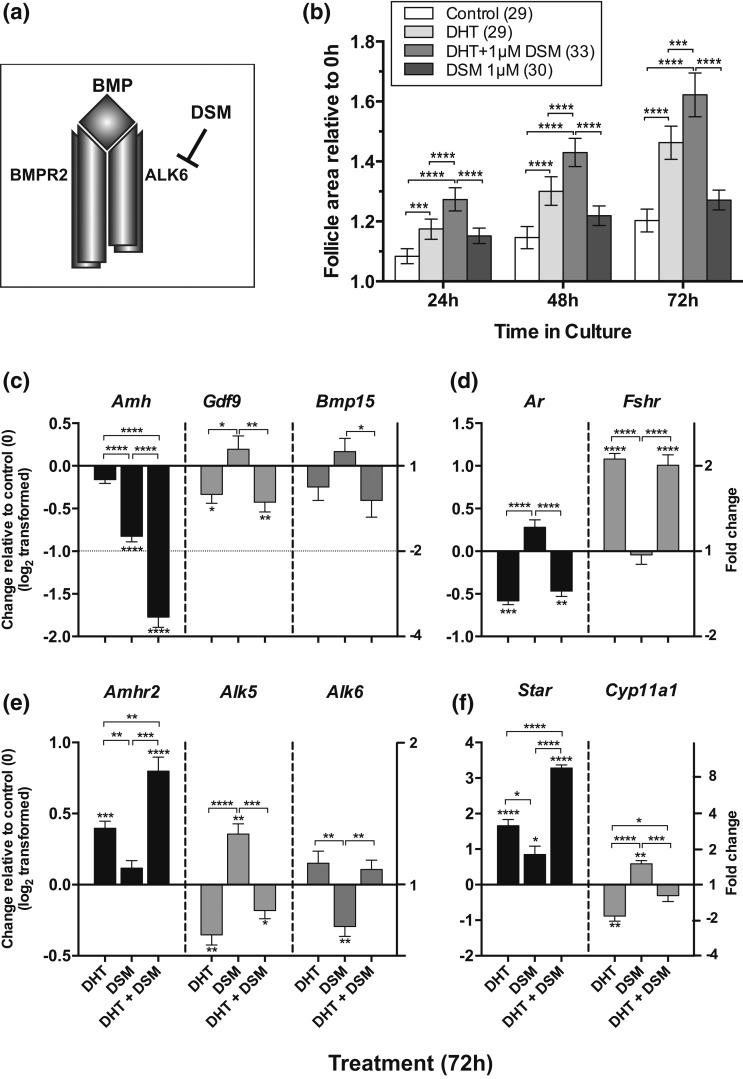
Effect of DSM (an inhibitor of Alk2, 3, and 6) on DHT-stimulated follicle growth *in vitro* for 72 hours. (a) TGF*β* receptor inhibition by DSM. (b) Isolated follicle growth in the presence and absence of DHT and/or DSM. Relative area of follicles in different treatments was compared at each time point using one-way ANOVA with a Tukey’s multiple comparisons test. *P < 0.05, **P < 0.01, ***P < 0.001, ****P < 00001. Values are mean and 95% confidence interval. Numbers in parentheses are numbers of follicles. (c–f) Effect of DHT alone, DSM alone, and DHT in combination with DSM on gene expression following 72 hours of culture. Data have been log_2_ transformed, and DHT, DSM, or DHT plus DSM treatment was compared with control samples (0) using an unpaired *t* test. Fold change is indicated in right-hand y axis. Asterisks by error bars indicate significant difference in expression from control, and differences between treatments are indicated by asterisks on horizontal lines. *P < 0.05, **P < 0.01, ***P < 0.001, ****P < 00001. Values are mean and SEM. (c) TGF*β* ligands. (d) Hormone receptors. (e) TGF*β* receptors. (f) Steroidogenic enzymes.

To explore the functional significance of androgen-induced changes in gene expression of TGF*β* ligands and their receptors, we examined the effects of the Alk2/3/6 inhibitor DSM (24) [Fig. 9(a)] on follicle growth and gene expression in the presence and absence of DHT. As shown in our previous study (23), DSM alone stimulated follicle growth at 24 hours (*P* < 0.05), but here we show that the response to DHT was significantly enhanced by the addition of DSM [Fig. 9(b)]. DSM did not, however, influence DHT-dependent antrum formation.

DSM alone resulted in reduction in expression of *Amh* after 72 hours in culture but with no significant effect on *Gdf9* [Fig. 9(c)]. However, DSM further enhanced the inhibitory effect of DHT on *Amh* without influencing DHT-induced *Gdf9* expression. Neither DSM alone nor DSM with DHT had an effect on expression of *Bmp15*. DSM alone had no effect on *Amhr2* but led to increased expression of *Alk5* and, as anticipated, reduction in *Alk6* [Fig. 9(e)]. The addition of DHT resulted in greater production of *Amhr2* than either DSM or DHT alone and reversed the effects of DSM on *Alk5* and *Alk6* [Fig. 9(e)].

There was no significant effect of DSM alone on *Ar* or *Fshr*, and DSM did not affect the response to DHT [Fig. 9(d)]. However, DSM alone enhanced expression of *Star* and further increased DHT-stimulated expression [Fig. 9(f)]. Interestingly, DSM alone led to an increase in *Cyp11a1* and reversed the inhibitory response to DHT [Fig. 9(f)].

## Discussion

The purpose of these studies was to examine the role of androgens in preantral follicle development to provide insight into the mechanism of aberrant early follicle development in human PCOS. Exposure to excess androgen has been postulated to underpin the probable developmental origin of PCOS and its adult manifestations of reproductive and metabolic dysfunction (14, 27, 28). Using a mouse model of preantral follicle development that has been well validated in our laboratory, we have shown that exposure to both DHT and testosterone have a plethora of effects on both follicle growth and gene expression of key molecules implicated in follicle function. As shown in previous studies of cultured isolated follicles from mouse ovary (1, 9), androgens stimulate follicle growth. This effect is mirrored in studies of prentral follicle development in the primate ovary following *in vivo* androgen treatment (29, 30). In addition to the increase of follicle diameter in response to androgens, our results show a significant increase in expression of the proliferation marker Ki67, whereas the expression of proapoptotic markers TUNEL and caspase was no different between treated and untreated follicles. Furthermore, there was a 10-fold increase of antrum formation, an index of accelerated growth, in the presence of DHT. Thus, although androgens have been shown to have proapoptotic effects in GCs of large antral follicles (10, 11), the predominant action of testosterone and DHT in preantral follicles appears to be stimulation of GC proliferation and net follicle growth.

In sections of juvenile mouse ovary, we found that AR was expressed in the cytoplasm of oocytes, GCs, and theca at all preantral stages, including primordial follicles. DHT treatment increased the overall expression of AR in cultured follicles and, importantly, increased nuclear localization of AR, as would be expected in relation to androgen-induced growth and differentiation. Increased expression of AR protein was interesting and paradoxical, as we have also demonstrated here that *Ar* messenger RNA is decreased by DHT treatment during culture.

With the exception of the specific experiment exploring the effect of DHT in the presence of FSH, all the follicles in this study were cultured without FSH. Use of the database allowed examination of the response of follicles of increasing size to DHT alone. Small follicles less than 130 µm in diameter grew more in the presence of DHT. This is in contrast to our observations of the effects of FSH on preantral follicle growth in culture, in that responsiveness to added FSH was greatest in follicles larger than 130 µm in diameter (26), leading us to conclude that follicles larger than 130 µm in diameter had a greater requirement for FSH. It may be that the lack of response to DHT in larger follicles in the current study is due to the need for FSH to grow *in vitro*.

An important observation is the positive interaction with FSH in terms of both follicle growth and gene expression. Both FSH, as previously shown (26), and DHT, independently, stimulated growth of preantral follicles in culture, but FSH increased the responsiveness to DHT in a dose-dependent manner. After 72 hours in culture, the optimum dose of FSH induced a 30% increase in follicle growth above that of DHT alone. The amplifying effect of androgens on FSH-stimulated steroidogenesis was described more than 20 years ago by Wang and Greenwald (4), and recently Sen and colleagues have shown that DHT has both antiapoptotic and proproliferative effects, the latter being mediated, at least in part, by increased FSH receptor expression (31). Here we confirm the effects of DHT on FSH receptor gene expression and, in addition, show that testosterone has a similar effect. It is not clear whether the increase in the expression of *Fshr* is due to increased expression per cell or to the increased number of cells in follicles cultured in the presence of androgens (or indeed both). The action of both androgens was reversed by the AR antagonist flutamide, indicating that the effect of testosterone was not likely to be attributable to its possible conversion to estradiol. The most significant effect of androgens on the steroidogenic pathway was an increase in expression of *Star*, which, of course, has a key role in making cholesterol available for steroid synthesis. Both DHT and FSH independently and interactively increased *Star* expression. Conversely, expression of *Cyp11a1* was suppressed by exposure to androgens, but these findings are in accord with observations in studies of the ovaries of day 90 fetal sheep whose mothers were treated with testosterone (18)

Very little has been reported to date on the interaction of androgens and the TGF*β* family of growth factors. There are isolated reports that TGF*β*1 inhibits androgen production by theca cells (32, 33), but, as far as we are aware, there have been no systematic studies of the effects of androgens on TGF*β* signaling. Our group has previously reported on expression and action of TGF*β* growth factors in isolated mouse preantral follicles (22, 23), and in the current study, we examined the interaction of androgen with TGF*β* ligands and their receptors. The most significant findings were inhibition by androgens of *Amh* and *Bmp15* expression. AMH has a well-described inhibitory action of preantral follicle growth (34), and BMP15 has a biphasic effect, initially stimulating growth but then leading to reduced growth rate and apoptosis in mouse preantral follicles after 48 hours in culture (23). In our studies, AMH expression was progressively reduced with time of exposure to androgens. Studies in sheep support this observation, as AMH protein expression is lower in large preantral follicles in 10- and 21-month-old prenatally testosterone-treated ewes compared with untreated controls (35). AMH and BMP15 activate receptor heterodimers that comprise the type 2 receptors AMHR2 and BMPR2, respectively, and the type1 receptor Alk6 that is used by both ligands. Androgen-stimulated follicle growth is accompanied by reduced expression not only of *Amh* and *Bmp15*, but also of *Amhr2* and *Bmpr2*. In addition, DSM, an inhibitor of Alk2/3/6, amplified DHT-stimulated follicle growth and further reduced expression of *Amh*. Taken together, these findings support the view that attenuation of the inhibitory effects of AMH and BMP15 play a role in the mechanism of androgen stimulation of preantral follicle development. There were also significant effects of DSM on the steroidogenic pathway in that, along with enhanced follicle growth, DSM amplified expression of key molecules involved early in the ovarian steroidogenic pathway (*Star* and *Cyp11a*) and modified the effects of DHT. This suggests a functional cross-talk between the TGF*β* system and the classic steroidogenic pathways. DSM has previously been described as an inhibitor of adenosine 5′-monophosphate kinase, but although we cannot rule out the possibility that this mechanism may play a part in the observed effects on follicle growth, its high selectivity for the BMP signaling pathway (24) makes the latter a more plausible target.

**Figure 10.**
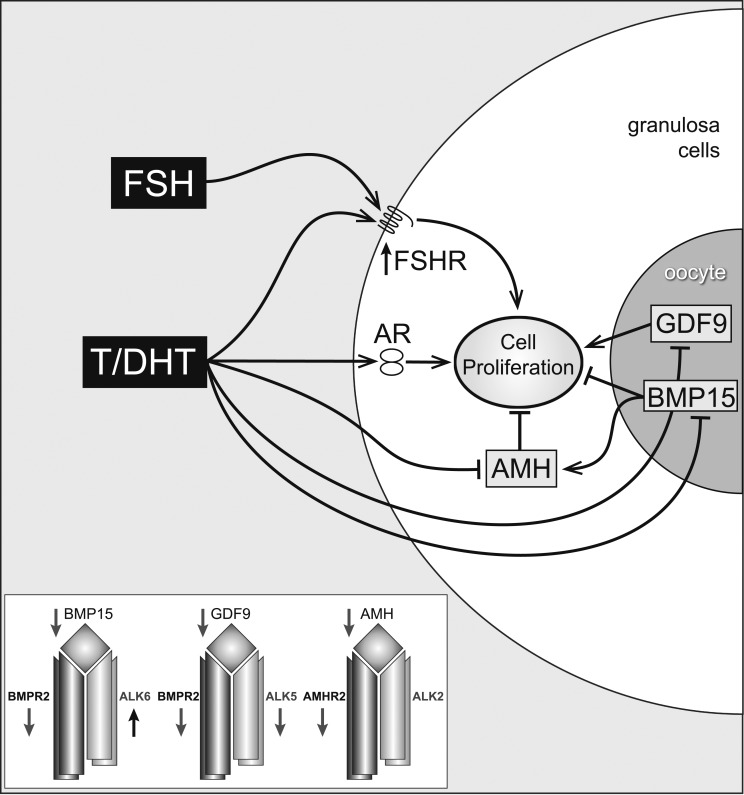
Putative pathways of androgen action on preantral follicle growth. Testosterone (T) or DHT act via the AR, causing a measurable increase in GC proliferation. This may be mediated directly, or indirectly, by increased FSHR (stimulating GC proliferation) or decreased AMH (reducing AMH inhibition). AMH can be further reduced by androgen-induced reduction of oocyte-specific BMP, which normally stimulates AMH levels. Inset box summarizes androgen-induced inhibition of TGF*β* ligands and type I and II TGF*β* receptors, with the exception of *Alk6*.

In addressing the issue of cross-talk between these pathways, one of the key findings in the current study was that, at 24 hours in culture, DHT treatment resulted in decreased expression of the oocyte-specific gene *Bmp15* (as well as decreased expression of *Amh* and *Amhr2*). These changes were accompanied by increased expression of *Star* and down-regulation of *Cyp11a*. In this context, it is striking that direct treatment of human granulosa lutein cells and ovine GCs with rhBMP15 results in opposite effects: increased *AMH*, *AMHR2*, and *CYP11A* and decreased *STAR* (19, 36, 37). This strongly suggests that BMP15 is a major regulator of both the rate-limiting steps of steroidogenesis and of AMH and its receptor and furthermore that DHT is having indirect actions on these genes via the consequent decreased levels of this oocyte-derived growth factor. Indeed, the important role of the oocyte in stimulating AMH expression was first proposed in 2004 in elegant studies using cocultures of isolated oocytes and preantral GCs from mice (38). The presence of one or more oocytes in the immediate vicinity of GCs increased AMH expression more than twofold. Furthermore, studies in our laboratory comparing AMH expression in intact preantral follicles and follicles lacking an oocyte have shown that the absence of an oocyte results in almost complete loss of AMH expression after 24 hours of culture (Mora and Fenwick, unpublished data). Thus, the effect of DHT (or testosterone) on follicle growth appears to involve a complex interaction with both FSH and the intraovarian TGF*β* system (Fig. 10). These androgens have a direct stimulatory effect on growth and also increase FSH responsiveness by up-regulation of FSH receptor expression. In addition, DHT and testosterone reduce expression of AMH both directly and by inhibition of the oocyte-derived TGF*β* growth factor BMP15. BMP15 has been shown to stimulate AMH production by GCs (and reduce expression of the growth-promoting GDF9 by the oocyte), hence reduction of BMP15 levels will contribute to reduced AMH levels. The net effect of androgens is therefore to contribute to GC proliferation by reversing the action of inhibitory TGF*β* growth factors that are produced by the oocyte and surrounding GCs. The current study has examined gene expression of these key ovarian factors, and it should, of course, be acknowledged that the changes observed do not necessarily imply similar effects on protein expression (as illustrated in this study with regard to AR gene and protein expression). Nevertheless, where we see evidence of functional changes (*e.g.*, androgen-dependent amplification of both *Fshr* gene expression in parallel with increased follicle growth rate), the association between gene and protein expression can easily be implied.

An intriguing effect of DHT on preantral follicle development was the precocious formation of an antrum, a feature that has also been reported in studies of PA sheep (39). Initially it was thought to be due to the stimulatory effects of DHT on follicle growth, with the expectation that antra would be present in the largest follicles. However, we found that there was no difference in the diameter of follicles with and without antra, suggesting that their appearance was due to effects of androgen on the expression of key genes, rather than achievement of a threshold diameter. Aquaporins facilitate water transport and are expressed in GCs (40), and testosterone stimulated the swelling of porcine GCs in hypotonic culture medium, an effect that was reversed by flutamide, suggesting that aquaporins are sensitive to androgen (41). A recent study by the same group confirmed that follicles from pigs that were exposed to flutamide *in utero* expressed less *Aqp5* messenger RNA and protein, demonstrating that androgen could potentially stimulate aquaporin expression leading to antrum formation (42).

Aberrant ovarian function associated with PCOS and hyperandrogenism has been well documented, and it has been suggested that “programming” of ovarian function by androgens plays a key part (27). This notion is supported by the results of studies of both rodent and large animal (sheep and monkey) models of PCOS that involve *in vivo* exposure to excess androgen (7, 19, 28, 43). Here, in an *ex vivo* model, we have examined the direct effects of androgens on follicle growth and gene expression and have uncovered pathways that are relevant to the abnormalities of follicle development that are characteristic of PCOS (14). The enhancement of FSH-activated follicle growth may well have a bearing not only on the accelerated growth of preantral follicles, but also on the precocious responsiveness to luteinizing hormone that has been observed in small antral follicles in ovaries of women with PCOS (14, 44). Intriguingly, androgen action also implicates the participation of key members of the TGF*β* superfamily and its receptors, and there is little doubt that these growth factors have a fundamental role in activation and maintenance of early follicle development.
